# Thin Film Formation Based on a Nanoporous Metal–Organic Framework by Layer-By-Layer Deposition

**DOI:** 10.1021/acsanm.4c04763

**Published:** 2024-11-01

**Authors:** Mario Fratschko, Tonghan Zhao, Jan C. Fischer, Oliver Werzer, Fabian Gasser, Ian A. Howard, Roland Resel

**Affiliations:** † Institute of Solid State Physics, Graz University of Technology, Graz 8010, Austria; ‡ Institute of Microstructure Technology, Karlsruhe Institute of Technology, Karlsruhe 76131, Germany; § Department Materials, Joanneum Research Forschungsgesellschaft mbH, Weiz 8160, Austria

**Keywords:** metal−organic framework, Cu_2_(bdc)_2_(dabco), layer-by-layer, thin film formation, X-ray diffraction, pole figures, texture analysis

## Abstract

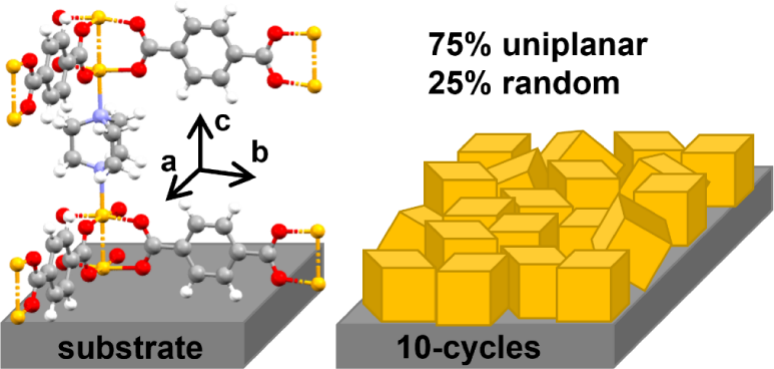

Understanding the structure of thin films is essential for successful applications of metal–organic frameworks (MOFs), such as low k-dielectrics in electronic devices. This study focuses on the thin film formation of the 3D nanoporous MOF Cu_2_(bdc)_2_(dabco). The thin films are prepared by a layer-by-layer technique with varying deposition cycles (1 to 50). Thin film morphologies and crystallographic properties were investigated using atomic force microscopy (AFM), Fourier transform infrared (FTIR) spectroscopy, and grazing-incidence X-ray diffraction (GIXD). AFM revealed an island growth (Volmer–Weber) with plate-like shaped islands. FTIR and GIXD revealed that Cu_2_(bdc)_2_(dabco) crystals form already during the first preparation cycle. The heights of the islands do not increase linearly with the number of deposition cycles, suggesting multiple growth stages. X-ray diffraction pole figures uncover a uniplanar texture of the Cu_2_(bdc)_2_(dabco) crystals, together with randomly oriented crystallites. The fraction of uniplanar oriented crystals increases with each deposition cycle, reaching a maximum of 75% at ten deposition cycles, simultaneously achieving complete substrate coverage. However, already at five cycles, an additional phase of randomly oriented copper-terephthalate (Cu_2_(bdc)) crystals appeared; this phase reaches a fraction of 22% at the largest film thickness (50 cycles). In summary, a detailed understanding of the thin film formation of an archetypal layer-pillar MOF is presented, elucidating how films grow in terms of their morphology and crystalline properties. Samples prepared by ten cycles show complete coverage of the substrate together with the highest degree of preferred crystal orientation. These results establish a deepened understanding of critical parameters for MOF thin film applications, such as complete substrate coverage and definition of the nanopores relative to the substrate surface.

## 1 Introduction

Metal–organic frameworks (MOFs) are highly porous materials with high chemical diversity, resulting in tunable properties,^1^ which makes them suitable for various applications, including gas storage and separation,^2^ drug delivery,^3^ sensing,^4^ and catalysis.^5,6^ The synthesis of MOFs is typically conducted under bulk conditions, e.g., solvothermal synthesis,^7^ resulting in polycrystalline materials formed by individual particles. However, applications in electrical devices require the use of thin films.^8−11^ The preparation of thin films requires specific techniques, including layer-by-layer growth,^12−14^ electrophoretic deposition,^15^ and heteroepitaxial growth,^16^ among others.^17^ Thin films prepared with these techniques have varying crystallite orientations, including random,^18^ biaxial,^16^ or uniplanar^19−21^ textures. Despite their benefits, these methods can be challenging due to issues like nonuniform coatings,^22^ time-consuming processes,^23,24^ or limited applicability to certain MOFs.^17^ Effective use of MOF thin films depends on controlling their structural alignment, as alignment of the MOF pores can significantly improve their performance for various applications.^25,26^ Consequently, an understanding of the thin film formation process is crucial and can be approached through a combination of techniques such as microscopy,^20,27,28^ infrared spectroscopy,^29^ and X-ray diffraction techniques.^21,28^ Of specific interest are the early growth stages of the thin film since these initial layers strongly influence the structural properties at later growth stages due to nucleation on the substrate surface.

**Scheme 1:**
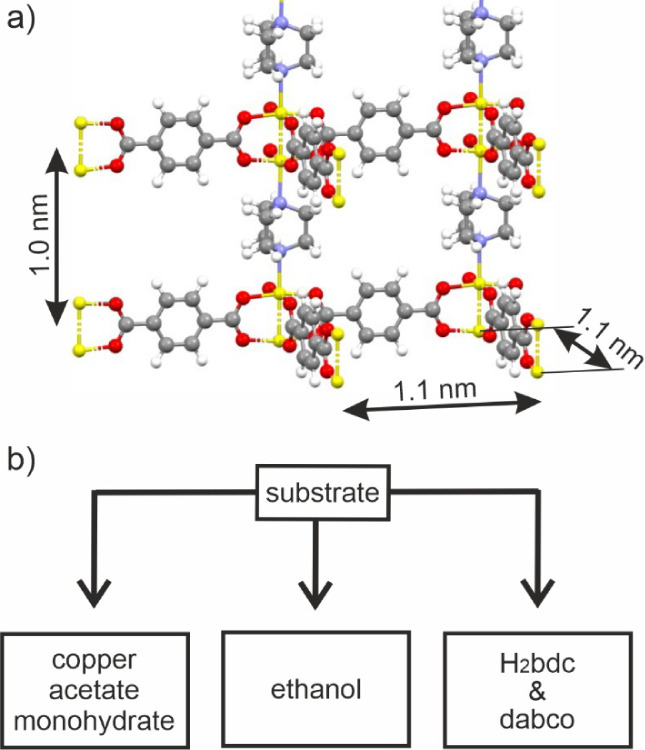
Internal Network Structure of the Metal–Organic Framework Cu_2_(bdc)_2_(dabco) a) Linker molecules bdc and dabco connecting the metal nodes formed by two copper atoms (yellow spheres); characteristic distances between the metal nodes are given. b) Layer-by-layer preparation by alternating treatment of a substrate with metal and linker precursor solutions with rinsing by ethanol between each step.

The MOF of interest in this study is Cu_2_(bdc)_2_(dabco), which is a pillar-layer MOF with an M_2_L_2_P crystal structure (M: metal ion, L: layer linker, and P: pillar linker).^30^ The MOF is composed of Cu paddlewheel nodes connected by benzene-1,4-dicarboxylate (bdc) linkers and 1,4-diazabicyclo[2.2.2]octane (dabco) pillars.^30,31^ 2D sheets are formed by the bdc linkers together with the Cu nodes, and a 3D network is formed by connecting the 2D sheets with dabco molecules. This molecular arrangement is shown in Scheme 1. The typical distances between the Cu nodes are ∼1 nm, which reveal the nanoporous nature of Cu_2_(bdc)_2_(dabco). These nanopores allow the desorption of hydrogen and aromatic molecules.^32,33^ A specific surface area of 1300 m^2^/g was found.^33^ In addition, a high potential as a catalyst was demonstrated.^31,34,35^ It is expected that the catalytic properties and the long-term stability are strongly dependent on the film thickness.^20^

Generally, the applicability of MOF thin films is related to the ability to obtain closed layers with full substrate coverage, as well as directed pore structures. Therefore, controlling the growth process in terms of the thin film morphology and crystallographic texture is essential in thin film preparation. Thin films of Cu_2_(bdc)_2_(dabco) were prepared in two different ways, using a layer-by-layer method^20,29,36,37^ and a ceramic-to-MOF approach.^16,38,39^

It could be demonstrated that the growth and orientation of the MOF crystallites can be controlled with specific methods involving self-assembled monolayers on a gold surface^29,40^ or a silicon surface by using different preparation protocols.^20,38^ Zhao et al. demonstrated that Zn_2_(bdc)_2_(dabco) grows with the (001) plane parallel to ethanol-rinsed Si(100) substrates using a layer-by-layer approach when a 2-cycle Cu_2_(bdc)_2_(dabco) seeding layer is used, while without the seeding layer, crystallite order is significantly reduced.^36^

The primary goal of this study is to uncover the evolution of the structural properties during the thin film growth process. The thin film morphology is linked to its crystallographic properties, providing a comprehensive understanding of the formation process during layer-by-layer deposition. This work should contribute to the pore structure implementation of MOF thin films, promoting their applicability.

## 2 Experimental Section

### 2.1 Thin Film Preparation

The layer-by-layer preparation of the thin film MOF Cu_2_(bdc)_2_(dabco) was based on the procedure presented by McCarthy et al.,^20^ wherein an ethanol-rinsed [100] silicon substrate is alternately exposed to two pump sequences alternating between a metal (Cu(CO_2_CH_3_)_2_·H_2_O) and a linker precursor solution (1,4-benzenedicarboxylic acid (H_2_bdc) and 1,4-diazabicyclo[2.2.2]octane (dabco) in ethanol) at a temperature of 62 °C with a rinsing procedure in between. A sketch of the schematic process is given in Scheme 1. After the final step, the samples were taken out of the ethanol and naturally dried at room temperature. More details about the sample preparation are given in the Supporting Information.

### 2.2 Atomic Force Microscopy

Atomic force microscopy (AFM) measurements were performed using Oxford Instruments Jupiter XR equipment. All measurements were performed in tapping mode using a Nanoworld Arrow NCR cantilever with a nominal frequency of 285 kHz. Image manipulation was performed using Gwyddion representing the data as height and phase contrast profiles.^41^ The height of the islands was determined by averaging the height distribution functions.

### 2.3 Infrared Spectroscopy

Fourier transform infrared spectroscopy (FTIR) was performed using a Bruker ALPHA spectrometer. Measurements were performed in transmission mode under ambient conditions, averaging 64 scans with a resolution of 4 cm^–1^ in the range between 400 and 4000 cm^–1^. Before measuring the samples, a background correction was applied by measuring a cleaned silicon substrate; nevertheless, some silicon peaks were present below wavenumbers of 1200 cm^–1^. Consequently, this range was excluded since MOF peaks cannot be distinguished from substrate peaks. OPUS software was utilized for data analysis and processing.^42^ The infrared spectra (*I*) shown are baseline-corrected by dividing the obtained intensity (*I*_raw_) by the baseline intensity (*I*_bl_) (determined with Origin software^43^): .

### 2.4 Grazing Incidence X-Ray Diffraction

Grazing incidence X-ray diffraction (GIXD) measurements were performed at the beamline XRD1, synchrotron Elettra, Trieste, using a wavelength of 1.40 Å, and the diffracted beam was detected with a Pilatus 2 M detector located 200 mm from the sample. Due to the low film thickness and thus the amount of material samples, up to 10 cycles were illuminated at an incidence angle of 0.2°, which is close to the critical angle of total external reflection. This increases the transmissivity of the diffracted beam. The samples with 20 and 50 cycles were investigated with an angle of incidence of 0.4° in order to reduce the foot print and therefore the peak width^44^ allowing the separation of experimentally overlapping peaks from different phases. The samples were rotated 360° during the GIXD measurement (rotating-GIXD) with individual diffraction images recorded for a defined rotation interval with an illumination time of 10 s. This procedure allows for the evaluation of pole figures for texture analysis. All experimental data were handled and transformed into reciprocal space using the software GIDVis.^45^ The observed intensities are available as a function of the components *q*_*x*_, *q*_y_, and *q*_*z*_ with a total scattering length of the scattering vector .^45^ Reciprocal space maps are plotted as an out-of-plane part (*q*_*z*_) and the in-plane part (*q*_*xy*_) with , obtained by the summation of the intensity for a complete sample rotation.

Before data analysis, intensity corrections were performed by averaging the intensity in a defined box in the diffuse region. This intensity was considered as the background and was further removed from the experimental raw data. Further corrections in terms of solid angle, pixel distance, detector efficiency, multiplicity, polarization, and Lorentz correction factor have been applied.

The GIXD data are used for phase identification by comparing qualitatively calculated and experimental peak patterns. Texture analysis is performed on the basis of pole figures, which represent the orientational distribution of the crystals within the thin film samples.^46^ Each pole figure is evaluated at a constant *q* with a specific data width of 0.005 Å^–1^ and is presented as a function of the azimuthal angle φ (range 0°–360°) and the polar radius ψ (range 0°–90°). A comparison between the experimental pole figures and a calculated stereogram (simulated by Stereopole,^47^ based on the crystal lattice of Cu_2_(bdc)_2_(dabco), is conducted. The obtained texture is classified according to the scheme of Heffelfinger and Burton.^48^

## 3 Results

The structural properties of the deposited films of Cu_2_(bdc)_2_(dabco) are investigated with several experimental techniques, including AFM for investigating the thin film morphology, FTIR is used to investigate the chemical composition, and rotating-GIXD is used to investigate the crystalline properties, including phase and texture analysis. The controlled preparation of the thin films by the layer-by-layer technique with different numbers of deposition cycles allows the investigation of the thin film structure as a function of thickness.

### 3.1 Thin Film Morphology

**Figure 1:**
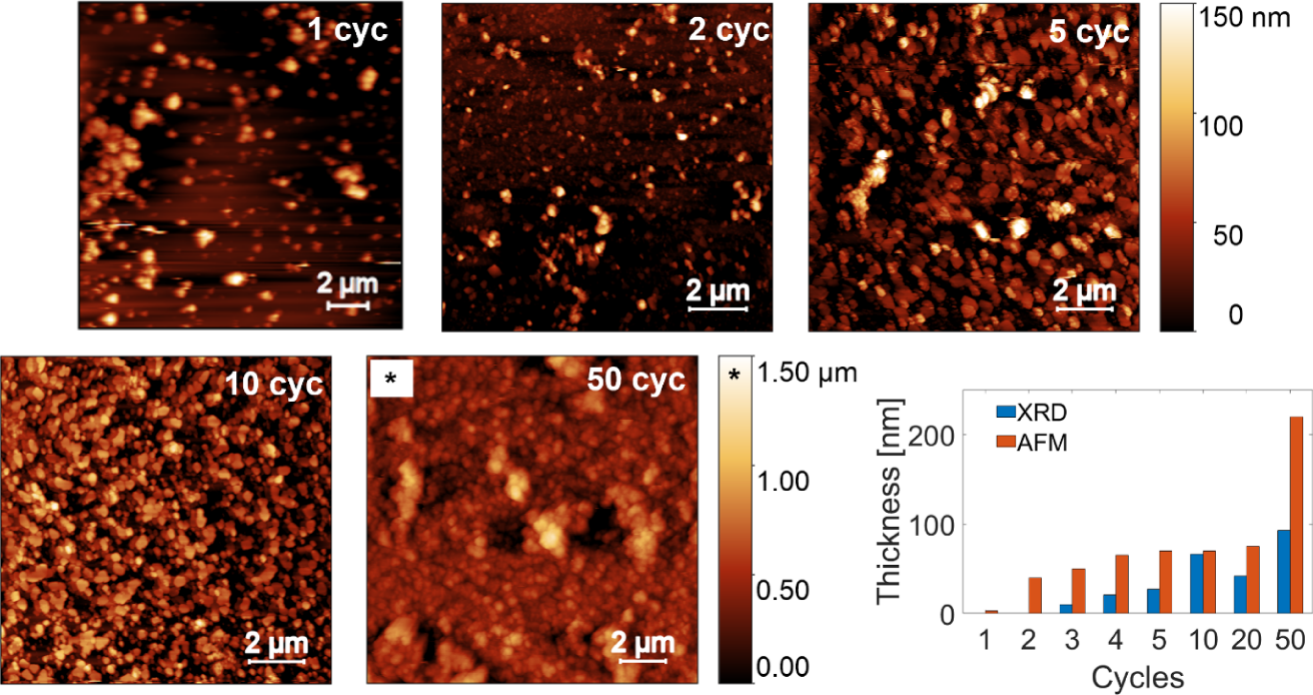
Atomic force microscope images of Cu_2_(bdc)_2_(dabco) prepared by different numbers of deposition cycles. The number of deposition cycles is given in the inset of each image. The *z*-height is presented at the same scale, except for the 50-cycle sample, whose specific *z*-scale is indicated with an asterisk. The average island heights and the vertical crystal sizes (from specular X-ray diffraction) are presented in a bar plot.

**Figure 2:**
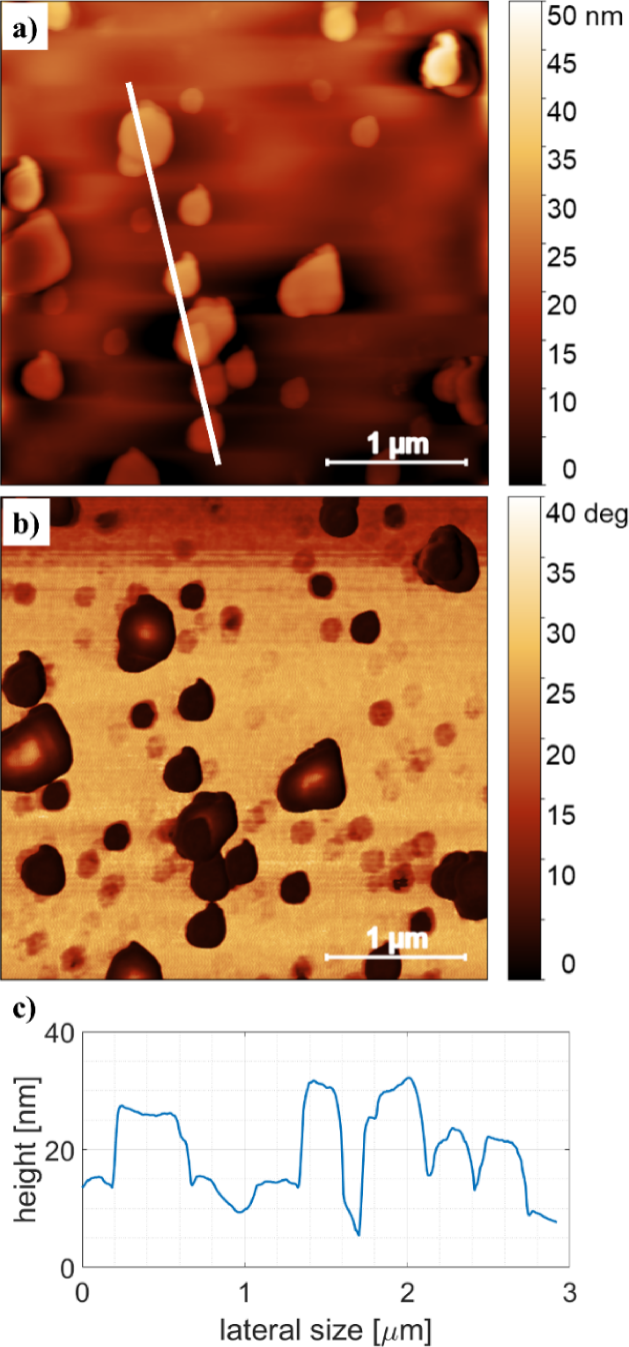
Detailed AFM image of the 1-cycle sample: a) a height image, b) the respective phase image, and c) a line scan (along the white line).

The evolution of the thin film morphology is shown in Figure 1 from AFM height images. Exemplary results of the samples prepared by 1, 2, 5, 10, and 50 cycles are shown, while the images of all samples studied are given in Figure S1. No deposition of the material could be observed for the 0.5-cycle sample. However, after one full cycle, individual islands with an average height of 15 nm and an average lateral size of 400 nm are present. Additionally, some agglomerations with lateral sizes of up to 1.7 μm are observed. An AFM image with a larger magnification height scan—depicted in Figure 2a—shows typical island heights in the range of 10 to 25 nm with lateral sizes in the range of 200 to 500 nm. The corresponding phase contrast image (Figure 2b) clearly distinguishes the islands from the surrounding area, suggesting a different material stiffness. The line scan (Figure 2c) reveals a plate-like morphology of the crystallites deposited in the first stage of the thin film formation.^19,36^ In general, the presence of isolated islands in the substrate is classified as an island growth mode (Volmer–Weber).^49^ A similar growth behavior is also reported for another type of MOF, e.g., HKUST-1.^27,50^

The 2-cycle sample shows an increased density of the islands as well as increased island height (Figure 1). Please note that the nominal film thickness could not be determined; therefore, the average island heights are obtained by height distribution functions based on AFM data. The 5-cycle sample exhibits an average island height of 70 nm while retaining the plate-like shape of the islands. At 10 deposition cycles, the average crystal height remains at 70 nm, and phase contrast imaging reveals full coverage of the substrate, as shown in Figure S2. It is important to note that the average height of the islands does not increase in a linear fashion with the number of deposition cycles. This phenomenon has been previously documented in the literature^51^ and is also illustrated in a bar diagram in Figure 1. The clear difference in the island height by AFM and crystal thickness by XRD is probably due to the limited order of the crystallites in the *z*-direction, which explains why XRD exhibits smaller crystal sizes.

### 3.2 Chemical Composition

**Figure 3:**
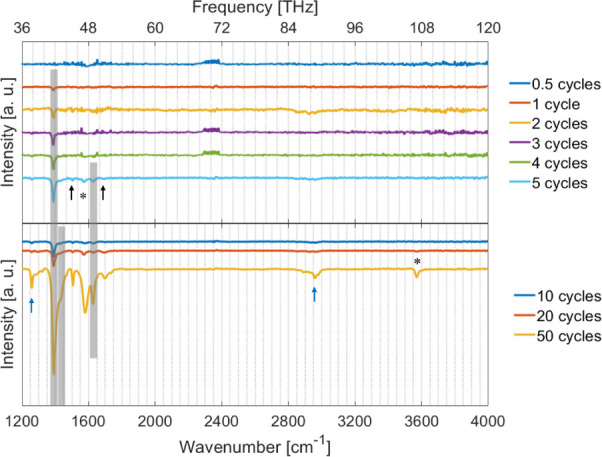
Infrared spectra of the layer-by-layer prepared Cu_2_(bdc)_2_(dabco) for different deposition cycles. The gray areas indicate the characteristic absorption peaks of Cu_2_(bdc)_2_(dabco), the asterisks (*) highlight characteristic peaks of the MOF Cu_2_(bdc), characteristic lines of bdc units are indicated by black arrows, and absorption peaks of the used solvent ethanol are marked with blue arrows.

Figure 3 shows the IR spectra for all deposition cycles, including the 0.5-cycle sample. The IR spectrum of the 0.5-cycle sample does not show any absorption peak. The first peak is observed in the 1-cycle sample at 1390 cm^–1^ (41.6 THz), marked by a gray vertical bar. Two distinct vibrational contributions are assigned to this peak. The first contribution is from the asymmetric stretching of the C–C atoms within the benzene ring, and the second contribution is from the scissoring mode of the dabco molecule.^30^ The 2-, 3-, and 4-cycle samples do not show any additional features; however, the absorbed peak intensities increase. In the 5-cycle sample, new peaks appear at 1505 cm^–1^ (45.1 THz), 1575 cm^–1^ (47.2 THz), 1629 cm^–1^ (48.8 THz), and 1690 cm^–1^ (50.6 THz). The peak at 1629 cm^–1^ (marked by a gray area) can clearly be attributed to the antisymmetric stretching of the COO^–^ of the bdc linker in the Cu_2_(bdc)_2_(dabco) structure.^30^ The other two peaks at 1505 cm^–1^ and 1575 cm^–1^ cannot be assigned to any of the Cu_2_(bdc)_2_(dabco) vibrational peaks but can be described with the vibrational modes of the bdc molecules and Cu_2_(bdc) crystals, respectively.^16,30,38^ With this assignment, the peak observed at 1575 cm^–1^ (marked with asterisks (*)) is the antisymmetric COO^–^ vibration of bdc in the Cu_2_(bdc) system, and the peak at 1505 cm^–1^ (marked with a black arrow) suggests a disordered region in the sample containing bdc^–2^ ions.^30^ The peak at 1690 cm^–1^ can be attributed to H_2_bdc (marked with a black arrow) as it is assigned by Falcaro et al.^16^

After 10 cycles, a new peak appears at 1433 cm^–1^ (marked by a gray bar), which can be attributed to the symmetric stretching of the COO^–^ of the bdc linker in the Cu_2_(bdc)_2_(dabco).^30^ At 20 cycles, a further feature appears at 3570 cm^–1^, marked with an asterisk, indicating the OH vibration in the Cu_2_(bdc) structure. At the deposition of 50 cycles, two further peaks appear at 1258 cm^–1^ and 2960 cm^–1^ (marked by blue arrows), which could arise from embedded ethanol from the sample preparation process, as these absorption peaks also emerge in the literature.^38,52^ The IR spectra suggest that besides Cu_2_(bdc)_2_(dabco) (peaks marked by gray areas), another MOF also develops in parallel, namely Cu_2_(bdc) (marked by asterisks (*)). Furthermore, the observed peak intensities give a first indication of the preferential orientation of the dabco linkers perpendicular to the substrate surface,^38^ which will be confirmed later through the X-ray diffraction studies.

### 3.3 Crystallographic Properties

#### 3.3.1 Qualitative Phase Analysis

**Figure 4:**
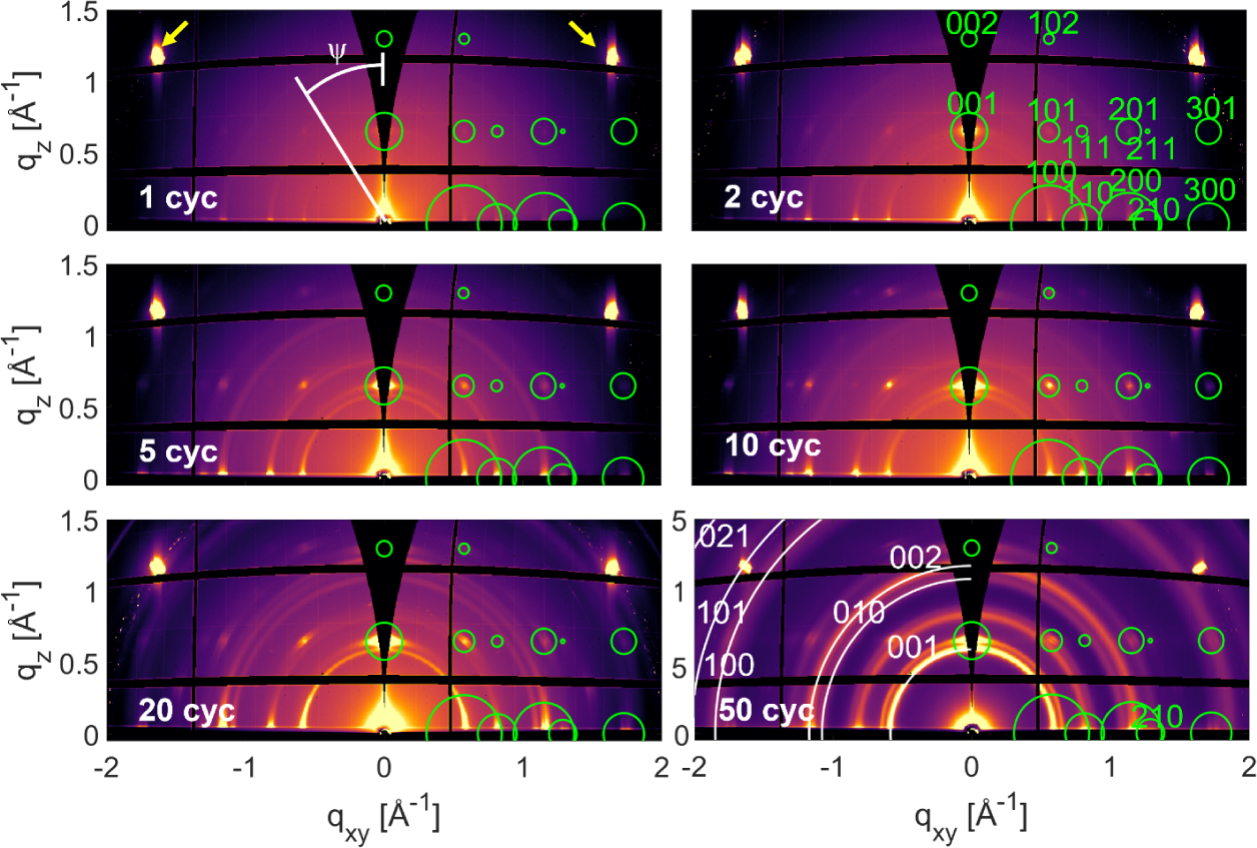
Reciprocal space maps of Cu_2_(bdc)_2_(dabco) thin film samples prepared by 1, 2, 5, 10, 20, and 50 deposition cycles; the respective number of cycles is indicated as an inset in each map. The green rings represent the calculated peak pattern of Cu_2_(bdc)_2_(dabco) with the center and the area of the circle indicating the peak position and the square of the structure factor, respectively. White rings plotted in the map of the 50-cycle sample indicate the Debye–Scherrer rings of the MOF Cu_2_(bdc).

The qualitative phase analysis is performed by comparing the experimental X-ray diffraction pattern with calculated ones based on the crystal structure of Cu_2_(bdc)_2_(dabco).^30^Figure 4 shows the reciprocal space maps of selected samples prepared by different deposition cycles, and the whole sample series is presented in Figure S3. The black areas in the reciprocal space maps are caused by blind spots of the detector, and the missing wedge around the origin of *qxy* (= 0 Å^–1^) represents experimentally inaccessible areas. The left side (*q*_*xy*_ < 0) as well as the right side (*q*_*xy*_ > 0) of the reciprocal space maps display identical diffraction information. For the sake of clarity, peak assignment is visualized only on one side. The strong diffraction peaks at *q*_*xy*_/*q*_*z*_ = ±1.632 Å^–1^/1.173 Å^–1^ arise from equivalent 111 Bragg peaks of the silicon substrate; both are marked by yellow arrows in the 1-cycle image.

The reciprocal space map of the 1-cycle sample shows weak but clearly distinct diffraction peaks at |*q*_*xy*_| = 0.575 Å^–1^, 0.815 Å^–1^, and 1.152 Å^–1^ with *q*_*z*_ ∼ 0 Å^–1^, at *q*_*xy*_/*q*_*z*_ = ∼0 Å^–1^/0.655 Å^–1^ and *q*_*xy*_/*q*_*z*_ = 0.575 Å^–1^/0.655 Å^–1^. These peaks can be indexed with the known crystal structure of Cu_2_(bdc)_2_(dabco).^30^ For visualization of the calculated peak positions and peak intensities, green circles are drawn where their center and area indicate the peak position and square of the structure factor of each individual calculated diffraction peak, respectively. Indexation is performed with the assumption that the (001) plane is parallel to the substrate surface. As the number of deposition cycles increases, the diffraction peaks become more intense, which is consistent with the findings of previous reports on a 20-cycle sample.^20,36^ Additional diffraction peaks appear at expected peak positions, accompanied by the presence of Debye–Scherrer rings, which are clearly visible in the 5-cycle sample and are located at the respective length *q* of the indexed peak positions.

In the 20-cycle sample, two new diffraction features are observed with Debye–Scherrer rings at *q* = 1.093 Å^–1^ and 1.852 Å^–1^, and both rings become more intense in the 50-cycle sample. These two features cannot be assigned to the Cu_2_(bdc)_2_(dabco) crystal structure. However, both rings can be related to the crystal structure of Cu_2_(bdc),^53^ the expected positions are plotted in Figure 4 as white rings for the 50-cycle sample. The expected peak positions fit exactly, suggesting the presence of additional MOF Cu_2_(bdc) appearing during Cu_2_(bdc)_2_(dabco) preparation, in conjunction with the earlier observations made by IR spectroscopy (Figure 3). Notably, there is a strong overlap between the 100 and 200 reflections of Cu_2_(bdc)_2_(dabco) with the 001 and 002 reflections of Cu_2_(bdc).

#### 3.3.2 Qualitative Texture Analysis

The thin film texture is determined by evaluating pole figures from the rotating-GIXD data. The pole figures are compared to simulated stereograms based on the MOF’s crystal structure. This approach enables the determination of the orientation distribution of the MOF crystallites within thin films.

**Figure 5:**
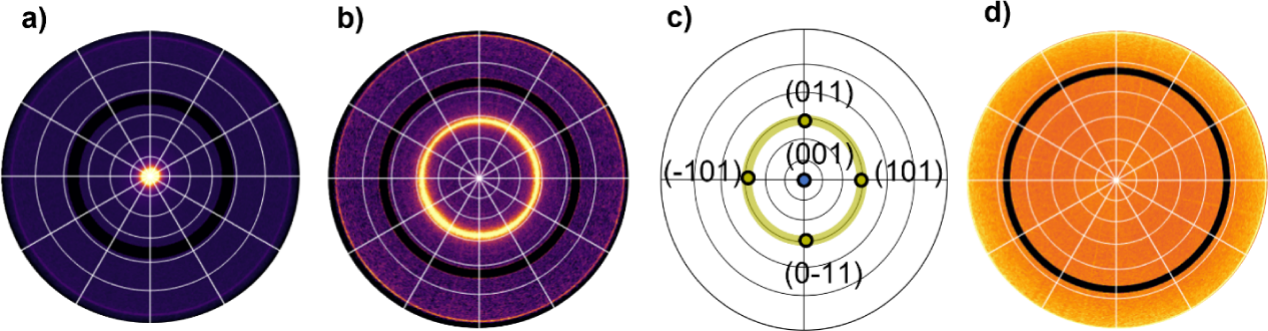
X-ray diffraction pole figures of a sample prepared in 20 cycles. a) The 001 peak taken at *q* = 0.65 Å^–1^, and b) the 101 peak taken at *q* = 0.86 Å^–1^ of Cu_2_(bdc)_2_(dabco). The concentric rings are in steps of Δψ = 15°. The calculated stereogram c) with the (001) pole at the center and the equivalent {101} poles at a defined polar radius, ψ = 41.7°. The yellow ring-like shape illustrates the position of the {101} poles by a crystal rotation perpendicular to the substrate surface. d) The pole figure of Cu_2_(bdc) taken at *q* = 1.08 Å^–1^ shows a homogeneous intensity distribution. The black rings in the pole figures are experimentally inaccessible areas.

The texture of Cu_2_(bdc)_2_(dabco) was analyzed for all deposition cycles from pole figures of the 001 and 101 Bragg peaks, based on two scattering vectors of *q* = 0.65 Å^–1^ and 0.86 Å^–1^, respectively. Figure 5a,b present both pole figures for the 20-cycle sample (pole figures of all samples are given in Figure S4). The 001-pole figure shows a central peak, indicating the alignment of the (001) plane parallel to the substrate, as already revealed by the indexation procedure used to explain the peak pattern of the reciprocal space maps in Figure 4. The 101-pole figure, however, shows a circular pattern. Both pole figures are compared with a calculated stereogram, presented in Figure 5c. Based on the crystal structure of Cu_2_(bdc)_2_(dabco), only the pole directions (or net plane normal) of the (001) plane and the equivalent {101} planes are selected. The comparison shows an exact overlap in terms of the polar radius of ψ = 0° for the (001) and ψ = 41.7° for the {101} poles. In contrast, the ring-like feature in the experimental 101 pole figure is clearly different from the simulated {101}-poles within the stereogram, which are at defined azimuthal angles (φ = 0°, 90°, 180°, and 270°). To obtain the same ring-like feature, a complete rotational freedom of the crystals perpendicular to the substrate surface must be considered. The texture analysis based on pole figures reveals that the (001) plane of the crystals is parallel to the substrate surface without any in-plane alignment of the Cu_2_(bdc)_2_(dabco) crystals, which is classified by Heffelfinger and Burton as a uniplanar texture.^48^ However, a second type of texture is present for the Cu_2_(bdc)_2_(dabco) crystallites. The observation of Debye–Scherrer rings (compare Figure 4) reveals the presence of randomly distributed crystals. The impact of random crystal orientations on the pole figures results in a constant intensity (or pole density) contribution spread over the complete pole figure. Randomly distributed crystals are classified as random textures.^48^

The coherent crystal size of the uniplanar-oriented crystallites was determined by a peak width analysis obtained by specular diffraction. The diffraction patterns are depicted in Figure S5, and the results are given in Figure 1. A continuous increase of the crystal size in the vertical direction is found, from 10 nm for the 3-cycle sample to 93 nm for the 50-cycle sample, based on the Scherrer equation.^54^

A texture analysis based on pole figures is also performed for the second crystalline phase present in our sample series, identified as Cu_2_(bdc). Figure 5d shows the pole figure taken at *q* = 1.08 Å^–1^ from the 20-cycle sample, and the result of the 50-cycle sample is depicted in Figure S6. The pole figure represents two independent Bragg peaks of Cu_2_(bdc) – namely 010 and 0–11 – which have to be monitored simultaneously due to the small difference in their characteristic *q*-values. No comparison to a calculated stereogram is required, since the pole figure displays a homogeneous intensity distribution over the whole orientation space, meaning that the crystals are randomly oriented.^48^

#### 3.3.3 Quantitative Texture Analysis

**Figure 6:**
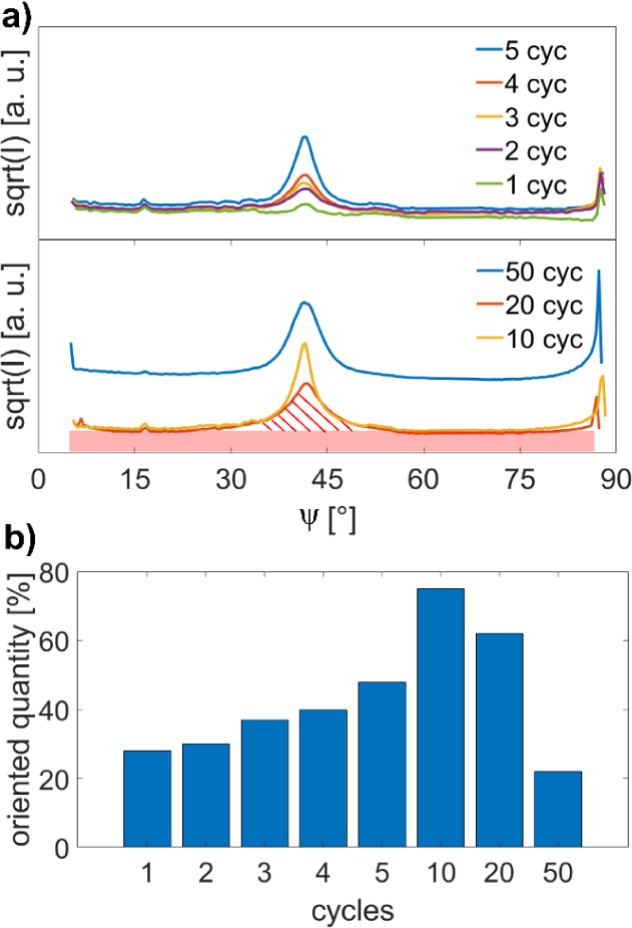
Quantitative analysis of Cu_2_(bdc)_2_(dabco) textures as a function of deposition cycles. a) Intensity profile of the 101 Bragg peak for the different deposition cycles. In case of the 20-cycle sample, the intensity contributions from the uniplanar texture (red shaded area) and the random texture (continuous red color) are explicitly marked. b) The fraction of the uniplanar-oriented crystals is presented for all deposition cycles in a histogram.

Once the specific textures have been identified, the fraction of the crystals associated with the two textures of Cu_2_(bdc)_2_(dabco) crystals is determined based on observed intensities. The radial intensity distribution of a Bragg peak along ψ is evaluated (for the definition of the angle ψ, see Figure 4, 1-cycle sample), which is achieved by summing a pole figure over the complete azimuthal angle φ in the range from 0° up to 360° (full rotation). Here, the pole figure of the 101 peak is chosen since the complete diffraction peak is present within the rotating GIXD experiment (see Figure 4). Before quantitatively analyzing the data, a further background correction must be performed in addition to the data correction described in the “Experimental Methods” section. For this correction, the background in the vicinity of both sides of the Bragg peak was averaged and subtracted from the raw data. Figure 6a shows the corrected intensity distribution of the summed radial pole figure of the 101 Bragg peak for the complete sample series. The enhanced intensity close to ψ = 90° arises due to the Yoneda peak and is not considered in the analysis. The intensity distribution is composed of contributions from two effects: First, the area below the peak arising from the uniplanar oriented crystals, and second, the area of constant intensity due to the randomly oriented crystals. These two contributions are exemplarily visualized for the 20-cycle sample: the contribution of the uniplanar oriented crystals is indicated with the shaded red lines, while the random contribution is represented by the red colored area. Notably, the mosaicity of uniplanar oriented crystallites, given by the peak widths in Figure 6a, does not change significantly, and values between 2.2° (10 cycles) and 4.8° (50 cycles) are found. The mosaicity values for all investigated samples are presented in Figure S7. Surprisingly, there is no clear change in the crystal mosaicities with the number of deposition cycles.

By determining the respective areas, we obtained the fractions of the individual crystal volumes. Figure 6b shows the fraction of uniplanar-oriented crystals in relation to the total volume of the crystals for all investigated samples. After the first deposition cycle, 28% of the crystals have a uniplanar texture, and 72% are randomly oriented. This fraction of crystals with a uniplanar texture increases for the 2, 3, 4, and 5 cycles to 30%, 37%, 40%, and 48%, until it reaches the highest value of 75% for the 10-cycle sample. At 20 deposition cycles, the proportion of oriented crystals starts to decrease, with 62% of the crystals exhibiting a uniplanar texture. For the 50-cycle sample, the texture exhibited further degradation, with the number of oriented crystals decreasing to only 22%.

#### 3.3.4 Quantitative Phase Analysis

**Figure 7:**
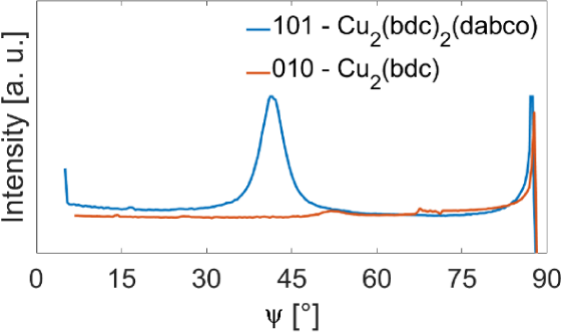
Intensity distributions of selected Bragg peaks of Cu_2_(bdc)_2_(dabco) and Cu_2_(bdc) present within the 50-cycle sample for quantitative phase analysis. The intensity distributions along Debye–Scherrer rings of the respective peaks are presented. The data are background corrected.

The quantity of the contributions of both MOFs, namely Cu_2_(bdc)_2_(dabco) and Cu_2_(bdc), which are present within the thin films, is determined by comparison of the corrected intensities of the two individual phases. This is achieved by first integrating the total intensity of the pole figures corresponding only to Cu_2_(bdc)_2_(dabco) and Cu_2_(bdc) at *q* = 0.86 Å^–1^ and 1.08 Å^–1^, respectively. Figure 7 depicts the intensity distribution along the characteristic Debye–Scherrer rings for the 50-cycle sample. The intensity of the (101) Bragg peaks of Cu_2_(bdc)_2_(dabco), as well as the summed intensity of the (010) and (0–11) Bragg peaks of Cu_2_(bdc), are displayed after background correction. The respective data without background correction are given in Figure S8.

In order to calculate the fraction of the two different phases, the intensity is corrected for both contributions in accordance with the methodology described in the literature.^44,55^ The comparison of the structure factors allows the individual contributions of each phase to be calculated for each cycle. For cycles 1 to 10, only Cu_2_(bdc)_2_(dabco) is present, as evidenced by the absence of the 010 and 0–11 peaks of Cu_2_(bdc) in Figure 4. The Cu_2_(bdc) structure begins to emerge after 20 cycles, representing 12% of the total thin film. At 50 cycles, 22% of Cu_2_(bdc) is present in the thin film, with only 78% of Cu_2_(bdc)_2_(dabco) remaining.

## 4 Discussion

The thin film growth of Cu_2_(bdc)_2_(dabco) prepared by the layer-by-layer method was investigated. AFM reveals a nonhomogeneous thin film growth, resulting from islands forming a plate-like shape, which is in good agreement with previous scanning electron microscopy investigations of a 20-cycle sample.^20^ The size distribution of the islands is between 200 and 500 nm for all deposition cycles, with some agglomerates having a lateral size of 1.7 μm, while the average height grows nonlinearly from an initial 30 nm (1 cycle) to 220 nm (50 cycles). Notably, in the 10-, 20-, and 50-cycle samples (Figures 1 and S1), the formation of additional small islands is observed, suggesting the ongoing nucleation of new islands, which is further supported by a comparable distribution of island sizes for all samples.

IR spectra reveal the successful preparation of Cu_2_(bdc)_2_(dabco), but also the growth of an additional Cu_2_(bdc) phase that appears in the sample prepared by 5 cycles (Figure 3). In the case of XRD investigations, the additional phase appears at a considerably later growth stage in the 20-cycle sample (Figure 4). Such a delayed visibility of an additional phase by X-ray diffraction is expected since a fraction of several mass% must be present for definitive verification.^56^ No clear explanation can be given for the formation of Cu_2_(bdc) at late thin film growth stages; however, nucleation of Cu_2_(bdc) and subsequent crystal growth is a required scenario. Such a model is supported by the observation of randomly distributed crystallites (Figure 5) and by the increasing fraction of Cu_2_(bdc) crystallites with the number of cycles. Literature suggests that the presence of water during the preparation process promotes the formation of Cu_2_(bdc) instead of Cu_2_(bdc)_2_(dabco).^57^ Even high humidity can cause the instability of Cu_2_(bdc)_2_(dabco) toward Cu_2_(bdc).^58^

**Figure 8:**
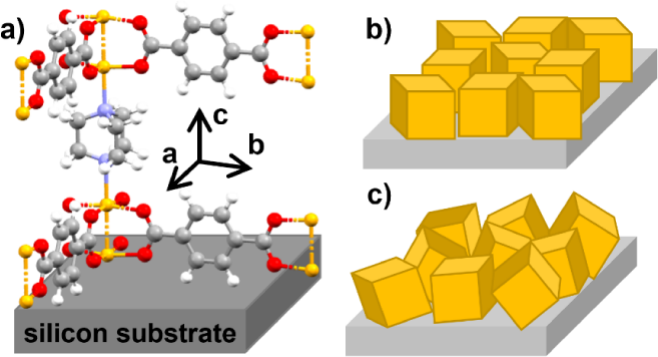
Alignment of the Cu_2_(bdc)_2_(dabco) MOF crystals relative to the substrate surface with a) a uniplanar texture revealing the dabco molecule perpendicular to the substrate surface. Schematic presentation of the two textures with b) the uniplanar texture and c) the random texture.

In the next step, the preferred orientation of the crystallites is discussed. It has already been reported that Cu_2_(bdc)_2_(dabco) crystallites form a uniplanar texture with the (001) plane parallel to the substrate surface.^30^ In this specific case, the planes formed by bdc linkers are parallel to the substrate surface, while the dabco linkers are normally aligned along the surface. This defines the orientation of the MOF structure relative to the substrate surface, as shown in Figure 8.

Additionally, randomly distributed crystallites are found, clearly detected by Debye–Scherrer rings (Figure 4). In the samples prepared by a small number of deposition cycles (1 cycle up to 10 cycles), the fraction of (001)-oriented crystallites increases with the number of cycles (Figure 6b). This observation suggests that in the initial growth stages, the nucleation process occurs in a rather directionless and unoriented way. However, with an increasing number of deposition cycles, the specific preferred orientation becomes more and more dominant until ten deposition cycles. Therefore, 75% of the total volume of the crystallites exhibit a (001) orientation relative to the substrate surface. One possibility to enhance the fraction of uniplanar crystals would be the use of specific growth layers deposited on the substrate surface prior to the MOF preparation.^59^

The small mosaicity of the (001) crystals and the weak dependence of the mosaicity on the number of deposition cycles (Figure S6) suggest that nucleation of the crystals with the preferred orientation exhibits a higher growth velocity than randomly oriented crystallites. This is also supported by the observation of the vertical crystal size of the preferred oriented crystallites, which increases continuously with the number of deposition cycles (Figure 1). Notably, the direction of crystal growth for the uniplanar oriented crystals is the crystallographic [001] direction, which is the direction of the dabco molecules. A limit of preferred growth is achieved in the 10-cycle sample. Discontinuities are observed in terms of crystal size (Figure 1), in the mosaicity (Figure S7), and in the fraction of preferred orientation (Figure 6b), where distributed crystallites show a higher fraction for the 20- and 50-cycle samples. Probably, randomly distributed Cu_2_(bdc) plays a role in impeding the dominant growth of the uniplanar Cu_2_(bdc)_2_(dabco) crystals.

## 5 Conclusions

The growth of Cu_2_(bdc)_2_(dabco) MOF thin films on silicon substrates is investigated by layer-by-layer deposition. Nine different samples were prepared with varying numbers of deposition cycles (0.5, 1, 2, 3, 4, 5, 10, 20, and 50), allowing the investigation of the thin film growth processes. AFM revealed separated islands on the substrate surface, which is clear evidence of an island-type thin film formation (Volmer–Weber). At ten deposition cycles, the substrate surface is completely covered by the MOF. With an increasing number of deposition cycles, the island height does not grow linearly, which indicates multiple mechanisms of thin film formation. GIXD reveals the spontaneous growth of two types of textures – uniplanar and random orientation of the Cu_2_(bdc)_2_(dabco) crystals. Both textures are already present in samples prepared by a few deposition cycles. Up to 10 deposition cycles, the fraction of uniplanar-oriented crystals increases more strongly than those of randomly distributed crystals. The dominant growth of the (001)-oriented crystallites is additionally suggested by the increase in vertical crystallite size and by their constant crystal mosaicities. At larger numbers of deposition cycles (20 and 50), the fraction of uniplanar crystals decreases from 75% (for the 10-cycle sample) to 62% and 22% for the 20- and 50-cycle samples, respectively. A second MOF phase starts to form after five deposition cycles, identified as Cu_2_(bdc). Random crystal orientation is found with a volume fraction of 22% determined for the 50-cycle sample. In summary, good levels of crystal orientation and film coverage were observed for the 10-cycle sample, whereas samples prepared by a lower number of deposition cycles do not fully cover the substrate surface. Thin films prepared by a larger number of deposition cycles show less pronounced orientation of the Cu_2_(bdc)_2_(dabco) crystallites, together with a larger fraction of Cu_2_(bdc) crystallites. The sample prepared by ten deposition cycles represents the best quality in terms of a closed thin film and a defined orientation of its nanopores.

## Supporting Information Available

Atomic force microscopy (AFM) images of all samples (Figure S1); AFM height and phase contrast of the 10-cycle sample (Figure S2); X-ray diffraction reciprocal space maps of thin films (Figure S3); X-ray diffraction pole figures of all samples (Figures S4 and S6); specular X-ray diffraction of all investigated thin films (Figure S5); histogram of the out-of-plane mosaicity of the complete sample series (Figure S7); radial intensity distributions of the 20- and 50-cycle samples (Figure S8) (PDF)
