# Supplementary material for: Thin Film Formation Based on a Nanoporous Metal–Organic Framework by Layer-By-Layer Deposition
Source: ACS Appl Nano Mater. 2024 Nov 1;7(22):25645–54. doi: 10.1021/acsanm.4c04763 (PMC11590058; doi:10.1021/acsanm.4c04763)
Supplement: Supplementary file 1 — an4c04763_si_001.pdf [file an4c04763_si_001.pdf]

Supporting Information

Thin Film Formation Based on a Nanoporous Metal-Organic Framework by Layer-by-Layer Deposition

*Mario Fratschko [(1)], Tonghan Zhao [(2)], Jan C. Fischer [(2)], Oliver Werzer [(3)], Fabian Gasser [(1)], Ian A. Howard [(2)], Roland Resel [(1)]**

(1) Institute of Solid State Physics, Graz University of Technology, Austria
(2) Institute of Microstructure Technology, Karlsruhe Institute of Technology, Germany
(3) Department MATERIALS, Joanneum Research Forschungsgesellschaft mbH, Weiz, Austria.
corresponding author*: roland.resel@tugraz.at*

CONTENT

The first chapter "Details on the sample preparation" complements the information on the sample preparation presented in the main paper.

In the following, supplementary experimental results are presented with complements the selected results presented in the paper. The morphologies of all samples are shown in Figure S1 by AFM height scans. A more detailed picture of the morphology is given for the 10-cycle sample in Figure S2. In addition to the height scan (Figure S2a), a phase scan (Figure S2b) is also presented. Upon zooming in (Figure S2c), it becomes evident that small dark spots marked with arrows indicate areas of uncovered substrate. These dark spots serve as a clear indication that the entire substrate is covered with the MOF.

Diffraction images of all samples are shown in Figure S3. Pole Figures are calculated from the diffraction images for all samples and are presented in Figure S4. The pole figures for the 1-, 2- and 50-cycle samples looks slightly different. The 1- and 2-cycle samples exhibit identical pole figures in comparison to the other samples, however, the diffracted intensity is very low (see Figure S3), therefore the signal to noise ratio is too high. In case of the 50-cycle sample an inhomogeneous ring-shape emerges which is a consequence of only half the substrate covered by the deposited film.Specular X-ray diffraction was performed for all samples to calculate the vertical crystal size of the crystals exhibiting uniplanar texture, which is shown in Figure S5. Measurements were performed with a PANalytical Empyrean diffractometer collecting information only of crystallographic planes parallel to the substrate surface. A sealed copper tube was used in combination with parallel beam mirrors and a beam mask (10 mm) for monochromatization (λ =

1.5418 Å) and parallelizing the X-ray beam, respectively. The diffracted beam was detected with a PixCel3D detector operating as a 1D line detector. The data are represented in the reciprocal space by calculating $q = \frac{4\pi}{\lambda} sin\theta$. The peak width Δq_z is used to determine the vertical size of the crystals by using the Scherrer formula, applied to the 001 Bragg peak of $Cu_2(bdc)_2(dabco)$.[1] Pole figures of the new appearing phase $Cu_2(bdc)$ for the 20- and 50-cycle samples are plotted in Figure S6. These pole figures are calculated for the two independent 010 and 011 Bragg peaks which are located at around q = 1.08 $Å^{-1}$. The mosaicity is not changing significantly with the number of deposition cycles as it is represented in Figure S7 as a bar plot. A visualization of the background correction is shown in Figure S8. Intensity distribution along Debye-Scherrer rings of the 101 Bragg peak of the $Cu_2(bdc)_2(dabco)$ phase and the 010 Bragg peak of the $Cu_2(bdc)$ phase are plotted for the samples with 20 and 50 deposition cycles.

Details on the Sample Preparation

All reagents and solvents were used as received or otherwise indicated. Copper acetate monohydrate ($Cu(CO_2CH_3)_2{\cdot}H_2O$, 98%, Alfa Aesar) was purchased from ThermoFisher (Kandel) GmbH. 1,4-Bibenzene dicarboxylic acid (bdc) was purchased from Merck KGaA. 1,4-diazabicyclo[2.2.2]octane (dabco, ≥ 99%) was purchased from Sigma-Aldrich GmbH. The preparation of the thin film MOF $Cu_2(bdc)_2(dabco)$ was based on the layer-by-layer synthesis presented by McCarthy et al.,[17] wherein, an ethanol-rinsed [100] silicon substrate is alternately treated in a heated metal and a heated linker precursor solution at a temperature of 62°C. The metal precursor solution was prepared by dissolving 1 mM of $Cu(CO_2CH_3)_2 \cdot H_2O$ in ethanol upon ultrasonication. Accordingly, for the linker precursor, 0.4 mM of 1,4-benzenedicarboxylic acid (H_2bdc) and 0.2 mM of dabco were dissolved together in ethanol and ultrasonicated until a transparent liquid was formed. The first step in the MOF growth process involved the treatment of the cleaned silicon substrate in the metal precursor solution for 15 min, followed by two rinsing cycles with pure ethanol, the first lasting for 0.5 min and the second for 4.5 min, with the exception of the first cycle. After rinsing, the substrate was exposed for 30 min to the linker precursor solution followed by the same rinsing procedure. The complete process was repeated 1, 2, 3, 4, 5, 10, 20 and 50 times (denoted as cycles) for different thin film samples, with a final step of ethanol rinsing. Subsequently, the samples were taken out of the ethanol and naturally dried at room temperature. In addition, one sample was prepared with treatment only with the metal precursor solution (0.5 cycles).

Experimental Results

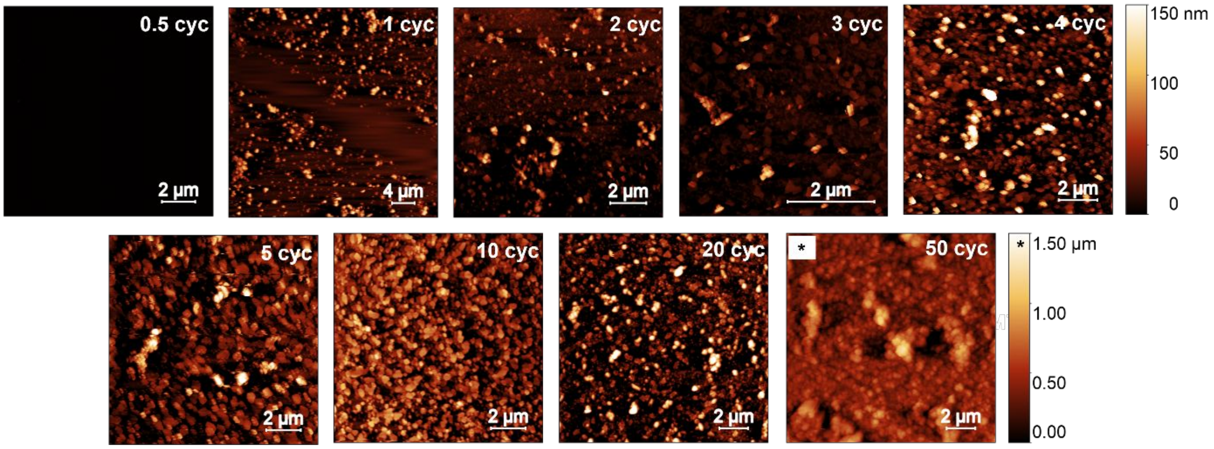

Figure S1. Atomic force microscope images showing the thin film morphology of $Cu_2(bdc)_2(dabco)$ of all samples, with the number of deposition cycles inserted in the image. The image for the 50-cycle sample shows a different z-scale indicated with an asterisk.

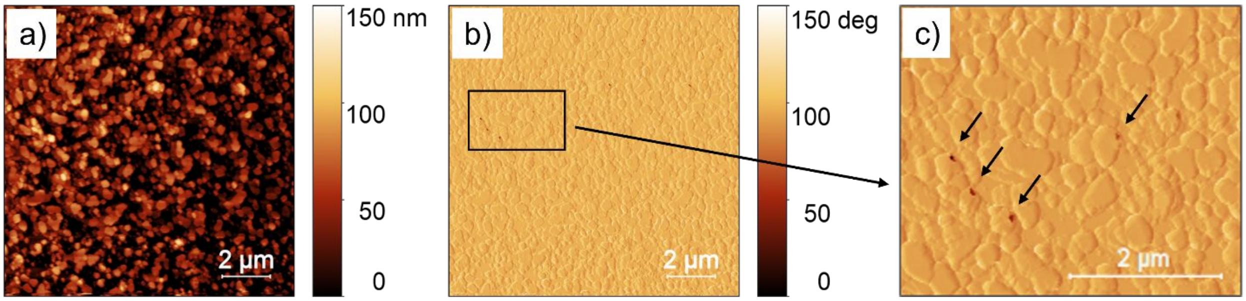

Figure S2. a) Height scan and b) phase contrast image of the 10-cycle sample. c) A detail of the phase contrast image reveals small spots (denoted by arrows) with material contrast due to uncovered substrate surface

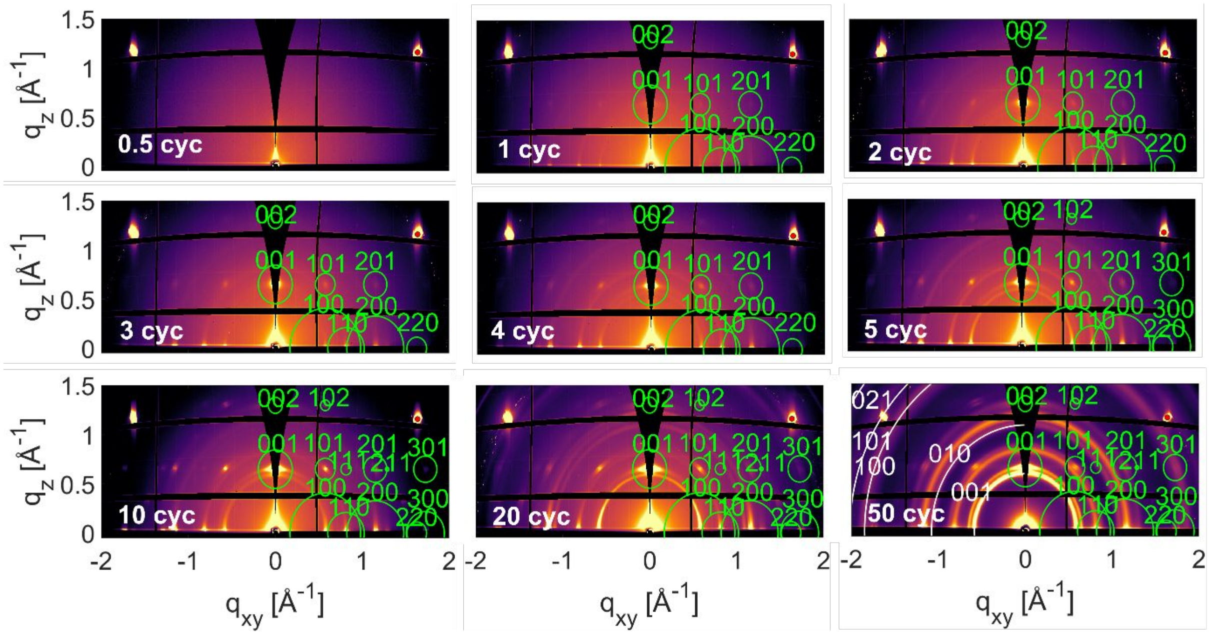

Figure S3. Reciprocal space maps of $Cu_2(bdc)_2(dabco)$ thin films. The green rings represent the calculated peak pattern of $Cu_2(bdc)_2(dabco)$ with the center of the cycle indicating the peak position and the radius of the cycle indicating the intensity. White rings plotted within the map of the 50-cycle sample indicates Debye-Scherrer rings of $Cu_2(bdc)$ visualizing only the peak positions.

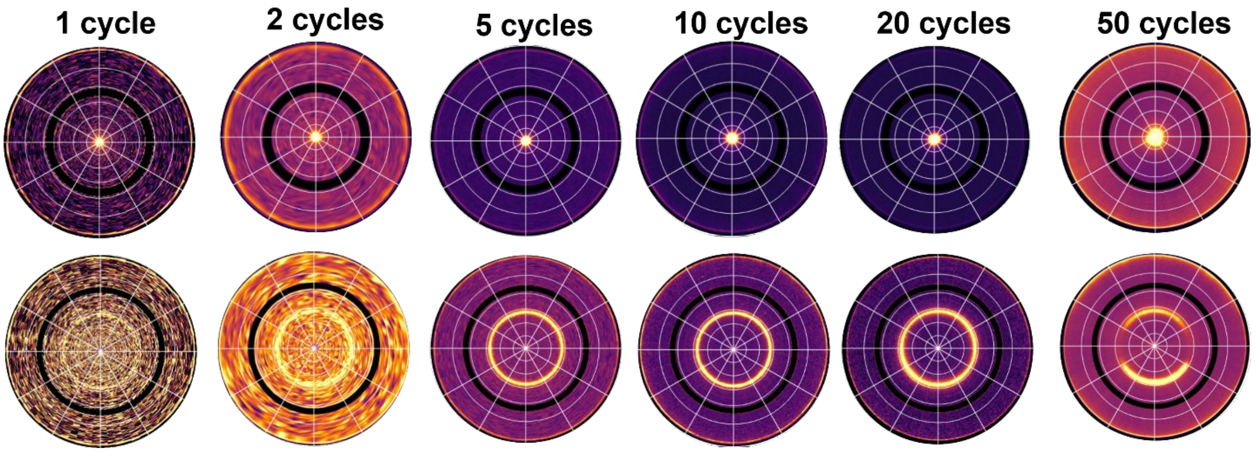

Figure S4. X-ray diffraction pole figures for all investigated $Cu_2(bdc)_2(dabco)$ samples of a) the 001-peak evaluated at q = 0.65 Å^{-1} and b) the {101}-peak series evaluated at q = 0.86 Å^{-1}. The inhomogeneous ring-shaped feature of the 101-pole figure for the 50-cycle sample is a consequence of the measurement geometry caused by half thin film coverage of the substrate.

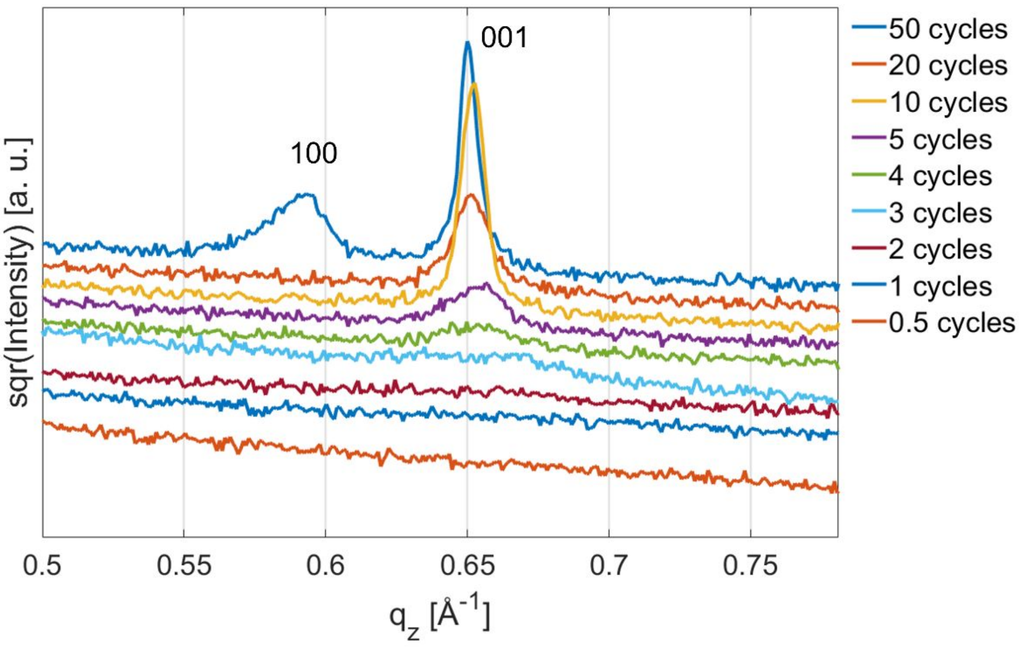

Figure S5. Specular X-ray diffraction pattern of all investigated $Cu_2(bdc)_2(dabco)$ thin films, plotted as a function of q_z. The Scherrer equation was employed at the 001 peak to determine the vertical crystal size.

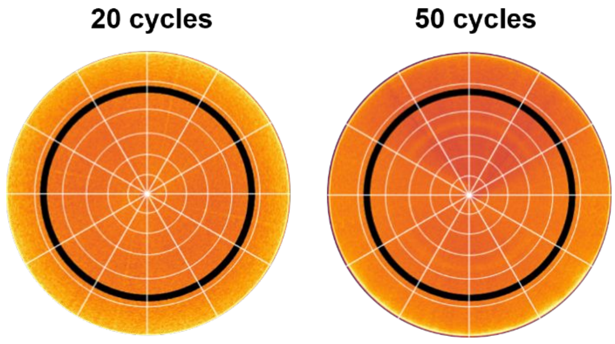

Figure S6. X-ray diffraction pole figures of $Cu_2(bdc)$ taken at $q = 1.08$ $Å^{-1}$ representing the poles of the 010 and 0-11 peaks for the 20- and 50-cycle samples.

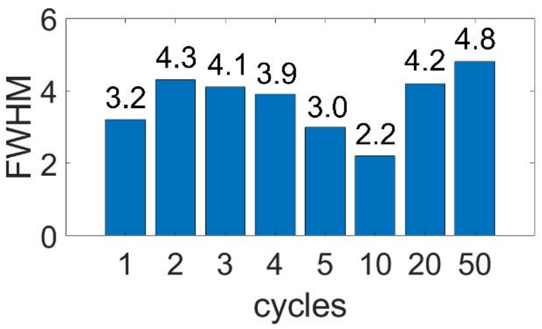

Figure S7. Histogram representation of the out-of-plane mosaicity of the complete sample series determined from the radial intensity distribution of the 101 Bragg peaks. The full width at half maximum (FWHM) of the 101 Bragg peaks are shown.

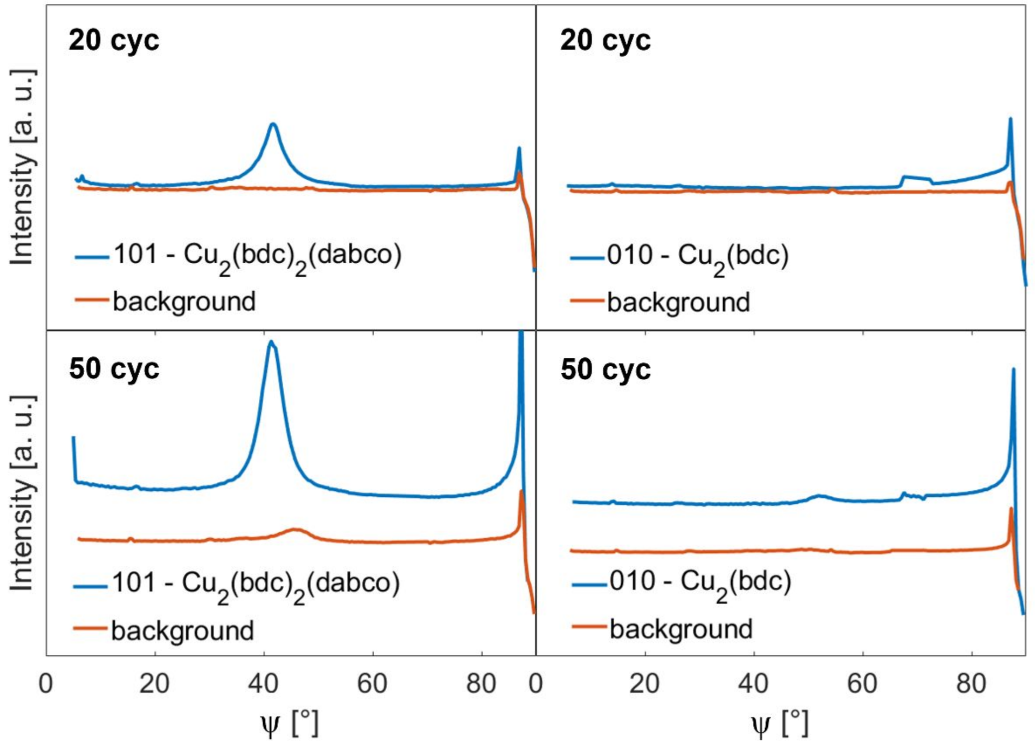

Figure S8. Intensity distribution along Debye-Scherrer rings of the 101 Bragg peak of the $Cu_2(bdc)_2(dabco)$ phase and the 010 Bragg peak of the $Cu_2(bdc)$ phase for samples with 20 and 50 deposition cycles. The data are presented at the same scale.

REFERENCES

(1) Patterson, A. L. The Scherrer Formula for X-Ray Particle Size Determination. *Phys. Rev.* **1939**, *56* (10), 978–982. https://doi.org/10.1103/PhysRev.56.978.
